# Targeting senescent cells in post-traumatic osteoarthritis: mechanisms, microenvironment remodeling, and translational prospects

**DOI:** 10.7717/peerj.20598

**Published:** 2026-03-16

**Authors:** Jipeng E, Qiang E, Guangsan Zhou

**Affiliations:** Suihua First Hospital, Suihua City, China

**Keywords:** Post-traumatic osteoarthritis, Cellular senescence, Senescence-associated secretory phenotype, Joint microenvironment, Senolytics, Cartilage degeneration, Inflammation, Tissue remodeling

## Abstract

**Background:**

Post-traumatic osteoarthritis (PTOA) progresses rapidly after joint injury and frequently affects young adults. Recent research has implicated senescent cells and their pro-inflammatory secretome as key contributors; however, the mechanisms linking trauma-induced senescence to cartilage degeneration remain poorly defined. This review synthesizes emerging evidence on senescence-targeted strategies in post-traumatic osteoarthritis and situates trauma-induced senescence within systemic aging and age-related osteoarthritis paradigms. Joint injury induces DNA damage and oxidative stress in chondrocytes and synovial cells, activating senescence-associated pathways (p53/p21, p16 INK4a). These senescent cells secrete inflammatory factors, proteases, and chemokines, collectively known as senescence-associated secretory phenotype (SASP), which accelerates cartilage degradation, subchondral bone remodeling, and cellular senescence. Unlike age-related osteoarthritis, PTOA is characterized by rapid and localized senescence following trauma. Pre-clinical studies have demonstrated that selectively eliminating senescent cells or inhibiting their SASP significantly reduces cartilage damage and the associated pain. Advanced therapeutic strategies utilizing targeted drug delivery systems, such as nanoparticles and gene therapy vectors, are emerging to specifically target senescent cells and to limit their adverse effects.

**Conclusion:**

Targeting cellular senescence is a promising disease-modifying strategy for PTOA treatment. Effective translation into clinical practice will require optimizing therapeutic delivery, determining intervention timing, and developing robust biomarkers to identify patients most likely to benefit.

## Introduction

Post-traumatic osteoarthritis (PTOA) accounts for approximately 12% of all symptomatic knee and hip OA cases worldwide; however, its incidence is rising faster than that of primary OA due to population aging, continued participation in high-energy sports, and increased motor vehicle use (Maia et al., 2023). Large cohort studies have estimated that up to 50% of anterior cruciate ligament injuries and 20% of tibial plateau fractures progress to radiographic PTOA within ten years despite contemporary fixation and rehabilitation protocols (Lohmander et al., 2007). The societal cost is substantial: in the United States alone, PTOA generates more than US$ 3 billion in direct treatment expenses and indirect productivity losses each year, with younger working-age adults bearing a disproportionate share (Hardenberg et al., 2022). Clinically, PTOA emerges earlier, advances more rapidly, and responds less favourably to conventional symptom-modifying drugs than age-related (primary) osteoarthritis, which is driven by cumulative micro- and macro-injury with innate-immune activation (Kraus et al., 2015). This therapeutic gap reflects a complex local milieu: focal cartilage loss often coexists with residual malalignment, ligamentous laxity, chronic low-grade synovitis, and obesity-linked meta-inflammation that perpetuate SASP signalling (Horváth et al., 2023). Together, these biomechanical and inflammatory insults continue to aggravate joint degeneration (Hunter, March & Chew, 2021). Current disease-modifying options remain experimental and joint-preserving osteotomies or arthroplasties in patients aged <50 years create long-term revision concerns, underscoring the urgent need for mechanistically targeted preventive and therapeutic strategies. Cellular senescence is a stress-induced state of stable cell cycle arrest, accompanied by profound metabolic, epigenetic, and secretory reprogramming. In articular cartilage and periarticular tissues, mechanical overload, DNA damage, and oxidative bursts after injury activate the p53–p21 and p16ˆINK4a–RB pathways, driving chondrocytes, synoviocytes, and osteoblast-lineage cells to senesce within days (Jeon et al., 2017). This secretome reinforces paracrine senescence, recruits innate immune cells, and accelerates extracellular matrix degradation, thereby linking an acute insult to chronic self-propagating joint deterioration (Coryell, Diekman & Loeser, 2021). Comprehensive reviews have synthesised the role of chondrocyte senescence in primary (‘age-related’) osteoarthritis (Huang & Cai, 2025), yet post-traumatic OA remains comparatively under-explored. Accumulating evidence shows that selective clearance or functional silencing of senescent cells (“senolytics” and “senomorphics”) alleviates pain and preserves cartilage in pre-clinical PTOA models, positioning senescence as a tractable therapeutic node (Dhanabalan et al., 2023; Jeon et al., 2017). This narrative review synthesizes current knowledge on trauma-induced cellular senescence in post-traumatic osteoarthritis (PTOA). We focus on three areas: (i) molecular and biomechanical triggers of senescence, (ii) SASP-mediated remodeling of the osteochondral microenvironment, and (iii) emerging therapeutic strategies for PTOA.

The review targets biomedical researchers and clinicians interested in osteoarthritis, musculoskeletal injury, and geroscience. By integrating mechanistic insights with translational advances, we aim to guide orthopedic scientists studying PTOA pathogenesis and support rheumatologists and translational researchers who seek senescence-targeted interventions for joint injuries.

### Literature search methodology

Databases and sources: We performed a comprehensive literature search using biomedical databases including PubMed, Web of Science, and Google Scholar. Additionally, reference lists of relevant articles were manually screened to identify further studies.

Search terms: Keywords used in various combinations included post-traumatic osteoarthritis, PTOA, cellular senescence, senescent cells, senescence-associated secretory phenotype, SASP, cartilage degeneration, synovitis, and senolytic therapy.

Selection criteria: We included peer-reviewed articles (original research and reviews) focusing on the mechanisms of senescence in osteoarthritis (especially trauma-induced contexts), the impact of senescent cells and SASP on joint tissues, and experimental therapies targeting senescent cells in musculoskeletal disease. Both *in vitro* and *in vivo* studies were considered. Articles were excluded if they were not in English, were single case reports unrelated to PTOA mechanisms or did not substantially address senescence or PTOA.

Timeframe: The primary literature search covered publications from approximately 2000 through April 2025, with an emphasis on the most recent decade of research to capture the latest advances. Earlier classic studies are referenced for foundational concepts.

Coverage and bias mitigation: To ensure comprehensive and unbiased coverage, we drew from multiple research groups and perspectives. We included studies with differing findings (*e.g.*, both positive and negative outcomes of senescence-targeted interventions) to provide a balanced overview. The search was updated through April 2025 to incorporate the latest available data, and multiple databases were used to reduce the risk of omitting relevant work. Because this review is narrative rather than systematic, quantitative tallies of records at each screening stage were not collected; instead, seminal and up-to-date studies that fulfilled the stated criteria were comprehensively discussed.

### Systemic ageing and age-related osteoarthritis: a geroscience framework

Chronological ageing is the single greatest risk factor for osteoarthritis, with population-based studies showing incidence curves that rise exponentially beyond the sixth decade (Loeser, Collins & Diekman, 2016). Unlike acute trauma, systemic ageing exerts multiorgan, low-grade inflammatory pressure (“inflamm-ageing”) driven by the progressive accrual of senescent cells in metabolic, immune and musculoskeletal tissues. Circulating SASP factors—including ANGPTL2, GDF-15 and cystatin-C—reach nanomolar concentrations in older adults and correlate with frailty and decreased bone-mineral density (Martín-Ruiz, Walaszczyk & von Zglinicki, 2022; Konstantinidi, Talage & Kennedy, 2017).

### Mechanisms of trauma-induced senescence

DNA damage–p53/p21–p16 axis: Genomic instability is one of the earliest molecular sequelae of joint trauma and activates the canonical p53/p21 checkpoint, which governs entry into cellular senescence. Following DNA strand breaks, phosphorylated p53 accumulates in the nuclei of damaged chondrocytes and drives the transcription of the cyclin-dependent kinase inhibitor p21, causing dual G_1/S and G_2/M arrest that freezes cell cycle progression while the repair machinery is mobilized. In addition to blocking mitosis, elevated p21 suppresses apoptotic cascades, allowing cells with persistent lesions to survive in a non-proliferative senescence-primed state (Palanivel et al., 2024). Therefore, mechanical overload in PTOA converts an acute genotoxic signal into chronic growth arrest that seeds the senescent cell population within the articular cartilage and amplifies early inflammatory signalling through DNA damage response-linked cytokine release in joint tissues. While p21 initiates the immediate checkpoint, prolonged or irreparable injury requires reinforcement of the p16ˆINK4a–RB axis. Sustained genotoxic or oxidative stress drives epigenetic de-repression of the CDKN2A locus, and accumulating p16 binds CDK4/6 to maintain RB in its hypophosphorylated form, locking damaged chondrocytes in permanent G_1 arrest even when the upstream p53 node is compromised (Liu et al., 2022; McHugh & Gil, 2018). By enforcing a stable senescent phenotype, p16 prevents aberrant proliferation, but simultaneously preserves cells that secrete matrix-degrading proteases and pro-inflammatory mediators, exacerbating joint degeneration. Oxidative bursts generated during hemarthrosis and the ensuing inflammatory cascade further entrench this checkpoint network by sustaining reactive oxygen species (ROS) signalling. ROS stabilizes p53, augments p21 transcription, and promotes CDKN2A hypomethylation, creating a feed-forward loop that promotes senescence (Zhang et al., 2021a; Zhang et al., 2021b; Muela-Zarzuela et al., 2024). Because chondrocytes possess limited antioxidant capacity, prolonged oxidative stress tilts the balance toward irreversible growth arrest, leading to clusters of senescent cells within the cartilage. These clusters release catabolic enzymes and inflammatory mediators that propagate extracellular matrix breakdown beyond the initial injury site, illustrating how trauma-induced DNA damage and oxidative stress translate mechanical injury into biochemical deterioration. Once established, senescence assumes a secretory phenotype that reshapes the periarticular milieu. Senescent chondrocytes and synoviocytes produce a SASP rich in interleukins, chemokines, and matrix metalloproteinases that recruit immune cells, intensify oxidative stress, degrade cartilage proteoglycans, accelerate joint aging, and foster chronic pain (Muela-Zarzuela et al., 2024). Complement activation provides an additional layer of modulation; for example, C5a signalling in the renal epithelia triggers epigenetic changes that amplify Wnt–β-catenin activity and hasten cellular senescence (Castellano et al., 2019). These findings underscore the conserved links between innate immunity and senescence pathways that may extend to the articular tissues exposed to trauma. In summary, trauma-induced senescence is driven by DNA damage and sustained checkpoint activation, and the resulting SASP-mediated paracrine signalling converts the focal injury into a degenerative cascade. This mechanistic insight highlights the need for targeted senolytic or senomorphic interventions in PTOA.

### Senescence-associated secretory phenotype and tissue remodelling

Senescent cells that accumulate after joint trauma secrete a complex blend of soluble and vesicular factors collectively known as the senescence-associated secretory phenotype (SASP). Proteomic catalogues have identified more than 40 cytokines, chemokines, growth modulators and matrix metalloproteinases, with IL-6, IL-8, CCL2 and MMP-13 among the most consistently elevated constituents (Goncalves et al., 2021). These mediators act cooperatively to reshape the joint micro-environment. Extracellular-matrix-degrading proteases intensify cartilage catabolism in concert with pro-inflammatory cytokines, whereas chemokines such as CCL2 recruit monocytes and sustain local inflammation in the injured joint (Jakhar & Crasta, 2019). Specific SASP components can serve as biomarkers. For example, the angiopoietin-like protein ANGPTL2 increases in senescent endothelial and stromal cells, reflecting the cellular stress in vascularized tissues relevant to osteochondral units (Thorin-Trescases et al., 2021). Hyperglycemia and mechanical overload further amplify SASP complexity by enhancing oxidative stress, which enriches the secretome with ROS-responsive mediators and accelerates catabolic signalling in post-traumatic cartilage (Zhang et al., 2019a; Zhang et al., 2019b). The composition of the SASP is not fixed but is shaped by intracellular and environmental cues that emerge immediately after tissue injury. Inducible cyclooxygenase-2 (COX-2) acts as a master modulator by driving prostaglandin E_2 synthesis, which reinforces IL-6 and MMP expression and blocks senescent chondrocytes in an autocrine inflammatory loop (Goncalves et al., 2021). Comparative analyses have shown that fibroblasts, synoviocytes, and chondrocytes exhibit distinct SASP repertoires, underscoring the influence of cell lineage and differentiation on the secretory output (Thorin-Trescases et al., 2021). Nutrient-sensing pathways provide an additional layer of control. For instance, the accumulation of branched-chain amino acids activates mTOR and broadens the pool of secreted catabolic factors (Liang et al., 2024b). ROS generated through NF-κB and IKK signalling further expands the SASP, and pharmacological IKK inhibition diminishes a panel of senescence markers in murine joints, illustrating the druggable nature of this regulatory axis (Zhang et al., 2020). Beyond its cell-intrinsic actions, SASP establishes a paracrine circuit that propagates senescence and modulates tissue regeneration after trauma. Conditioned media from radiation-induced osteocytes impair the osteogenic commitment of bone marrow mesenchymal stem cells *via* elevated IL-6 and CXCL1, thereby limiting the repair of subchondral bone (Xu et al., 2021). Similar intercellular crosstalk appears in the ageing skeleton, where a “bone SASP” enriched in GDF-15 and IGFBP-2 skews progenitor differentiation toward adipogenesis and undermines mechanical integrity (Fang, Liu & Wan, 2023). Temporal modelling indicates that the SASP evolves in phases: an initial wave of growth factors aids debris clearance, followed by a sustained influx of profibrotic mediators that can entrench chronic inflammation if resolution fails (Chandrasegaran, Sluka & Shanley, 2025). These dynamics imply that microenvironmental signals in the early post-injury window determine whether the senescence cascade remains transiently adaptive or shifts toward maladaptive chronic degeneration. Persistent exposure to the SASP drives a low-grade inflammatory milieu, often labelled as “inflammaging,” and contributes to systemic manifestations of musculoskeletal decline. Elevated ANGPTL2, GDF-15, and cystatin-C levels correlate with frailty indices and reduced bone mineral density in older adults, linking circulating SASP factors to multitissue dysfunction (Evans et al., 2024). In murine models, estrogen deficiency and cellular senescence independently accelerate trabecular bone loss, and combined exposure to both insults magnifies cortical porosity by approximately 25%, highlighting the synergistic damage associated with chronic SASP signalling (Farr et al., 2019). Senescent macrophages further intensify joint destruction through NLRC4-mediated IL-1β release, a mechanism amplified under metabolic stress such as hyperglycemia (Nie et al., 2019a; Nie et al., 2019b). Collectively, these findings suggest that SASP is a central amplifier of trauma-induced aging pathways in PTOA and provides a rationale for targeting upstream regulators or key SASP mediators as a therapeutic approach.

### Cartilage

Articular cartilage relies on a densely interlaced network of type II collagen and aggrecan to resist the compressive and shear forces. With advancing age, this matrix gradually loses resilience, as the equilibrium between synthesis and catabolism drifts toward degradation (Fujii et al., 2022). This shift reflects the upregulation of matrix-degrading enzymes that progressively fragment collagen fibrils and proteoglycan aggregates, compromising the tensile strength and fluid load support (Ashruf & Ansari, 2022). In experimental models, glycosaminoglycan content decreases by nearly two-fifths within weeks of surgically induced PTOA, mirroring the declines seen in aged human cartilage and underscoring how trauma can accelerate senescence-like molecular patterns (Fujii et al., 2022). Chondrocytes exposed to inflammatory cytokines (*e.g.*, IL-1β) rapidly amplify the transcription of MMP-13, which is mainly responsible for cleaving type II collagen (Ding et al., 2020). The broad substrate range of MMP-13 allows it to digest collagen and aggrecan alike, coupling structural failure with the loss of osmotic swelling pressure. Notably, elevated MMP activity (see Table 1) appears even in macroscopically intact cartilage adjacent to injury sites, implying a senescence-like secretory phenotype that spreads beyond visible lesions (Fujii et al., 2022). Such a spatial spread supports the concept that low-grade inflammation orchestrates joint-wide remodelling and highlights the potential value of silencing cytokine-driven MMP expression to preserve cartilage.

### Synovium: Inflammatory cascade and fibroblast senescence

The synovium undergoes age-related shifts that set the stage for an enduring inflammatory milieu in the osteoarthritic joints. Activated synovial fibroblasts release IL-1β, IL-6, and TNF-α at several-fold higher concentrations than those in non-arthritic tissues, recruiting macrophages that further intensify cytokine production (Li et al., 2022b; Chen et al., 2020a; Chen et al., 2020b; Han et al., 2020; Weng et al., 2021; Shi et al., 2023). This cytokine surge is accompanied by an increase in matrix metalloproteinases, such as MMP-3 and MMP-13, enzymes that degrade type II collagen and aggrecan and accelerate cartilage erosion (Li et al., 2022a; Han et al., 2020). Consequently, the synovial lining transitions from a lubricating membrane to a key driver of catabolic signalling, illustrating how tissue-specific aging remodels the joint microenvironment to favor degeneration. Persistent exposure to inflammatory mediators forces synovial fibroblasts into a state of cellular senescence, which is characterized by irreversible growth arrest and heightened secretory activity (Carr et al., 2020; Han et al., 2020). Senescent fibroblasts continue to expel cytokines and proteases that trigger their arrest—including IL-6, IL-8, MMP-3 and MMP-13, which sustain a cycle of tissue damage. Elevated TNF-α and IL-1β levels not only maintain the senescent phenotype but also disrupt local DNA-damage checkpoints, allowing senescent cells to accumulate with age (Salman et al., 2023; Han et al., 2020). Joints with a higher burden of senescent fibroblasts develop osteophytes more rapidly, exhibit thicker fibrotic synovium, and show greater pain behaviors than age-matched controls, underscoring the clinical significance of fibroblast senescence in PTOA.

**Table 1:** Summary of key SASP components and inflammatory mediators implicated in post-traumatic osteoarthritis (PTOA).

**SASP class**	**Representative mediators**	**Principal effects in PTOA**	**Key references**
Pro-inflammatory cytokines	IL-6, IL-1β, TNF-α	Activate NF-κB / MAPK signalling; amplify catabolic gene expression	Loeser, Collins & Diekman (2016) and Goncalves et al. (2021)
Chemokines	CCL2, CCL5, CXCL8	Recruit monocytes/macrophages; sustain low-grade joint inflammation	Chambers et al. (2021)
Matrix-degrading proteases	MMP-13, ADAMTS-5	Cleave type II collagen & aggrecan; MMP activity remains elevated even in macroscopically intact cartilage	Nie et al. (2019a) and Nie et al. (2019b)
Growth factors	ANGPTL2, GDF-15	Promote angiogenesis and bone remodelling; correlate with bone-density loss	Konstantinidi, Talage & Kennedy (2017)
Extracellular vesicles / microRNAs	miR-34a-enriched EVs	Mediate paracrine senescence; expand senescent cell pool	Basisty et al. (2020)

### Subchondral bone: imbalance between bone remodelling and angiogenesis

The subchondral bone plate is a dynamic interface where bone remodelling and angiogenesis are tightly coupled. Osteoclast-mediated resorption releases pro-angiogenic signals, whereas osteoblasts rely on capillary-borne oxygen, minerals, and vascular endothelial growth factor (VEGF) to sustain osteogenesis (Peng et al., 2020; Zhao & Xie, 2020). A specialized capillary subtype, the CD31ˆhiEmcnˆhi “type H” vessel, coordinates this bidirectional crosstalk and constitutes approximately one-third of the metaphyseal microvasculature in healthy young bones (Sivan, De Angelis & Kusumbe, 2019; Zhang, Pan & Jing, 2020). These vessels secrete angiocrine cues that expand osteoprogenitors and accelerate matrix deposition, ensuring that vascular maturation proceeds in lockstep with new bone formation. VEGF and platelet-derived growth factor-BB (PDGF-BB) are key molecular links in this loop as both stimulate endothelial proliferation and attract Runx2 + osteoprogenitors to perivascular niches, reinforcing the structural integrity of the subchondral plate during growth and repair (Jiang, Sun & Huang, 2022; Zhang, Pan & Jing, 2020). Physiological aging skews this fine-tuned relationship. Longitudinal imaging has shown that the volume fraction of type H vessels decreases from approximately 32% in young adults to <14% in the elderly, paralleling a marked reduction in osteoblast precursors and trabecular bone volume (Zhang, Pan & Jing, 2020). Endothelial expression of hypoxia-inducible factor-1α and Notch ligands also declines, diminishing the osteogenic angiocrine milieu required for balanced remodelling (Chen et al., 2021). The resulting hypovascular microenvironment compromises nutrient delivery and waste removal, slows matrix mineralization, and allows osteoclastic resorption to outpace bone formation. At the cellular level, aged pre-osteoclasts secrete less PDGF-BB, undermining the recruitment of endothelial progenitors required to replenish the capillary bed (Peng et al., 2020). Consequently, the subchondral plate becomes thinner and more porous, rendering the joint susceptible to post-traumatic degeneration when mechanical loads suddenly shift toward weakened osteochondral regions.

### Inter-tissue SASP crosstalk

Senescence-associated secretory factors function as multicellular signalling hubs within the joint. Proteomic mapping has revealed that more than 200 secreted proteins accompany the canonical SASP cytokines, giving each tissue a distinctive inflammatory fingerprint (Basisty et al., 2020). This biochemical dialogue causes otherwise separate tissues to act as a single inflammatory unit that is primed for accelerated degeneration. When SASP mediators persist long after acute injury, they push the joint toward chronic inflammation, mirroring ageing tissues in which sustained IL-6 signalling drives catabolic gene expression (Muñoz et al., 2019). Even low nanomolar concentrations of SASP cytokines can amplify NF-κB activity in stromal cells, locking them into a self-perpetuating prosenescent loop (Nacarelli et al., 2020). Conversely, the timely clearance of senescent cells by immune effectors can restore homeostasis, underscoring the context-dependent balance between protective and deleterious SASP actions (Wang, Lankhorst & Bernards, 2022). Metabolic cues add a further layer: diminished NAD^+^ levels in post-injury cartilage correlate with heightened NF-κB output, intensifying the secretion of IL-6, CCL2, and other SASP mediators that diffuse from cartilage to synovium (Nacarelli et al., 2019). In addition to these signals, SASP factors recruit macrophages, whose phenotypes evolve over time. Early after injury, they aid debris clearance, but prolonged exposure polarizes them toward a tissue-degrading state (Elder & Emmerson, 2020; Wang, Lankhorst & Bernards, 2022). This dynamic crosstalk between senescent, immune, and structural cells shapes the PTOA trajectory. Single-cell RNA sequencing further revealed that the SASP is not uniform: senescent chondrocytes upregulate metalloproteinase-linked chemokines, whereas senescent synovial fibroblasts preferentially secrete growth factors (Cherry et al., 2022). Such heterogeneity affects the magnitude of joint inflammation and influences drug responsiveness; for example, cells with high HMGB1 expression respond differently to IL-6 blockade than low-expressing counterparts (Xiao et al., 2024). Mapping these signatures could refine senolytic or SASP-modulating strategies by matching the interventions to the dominant communication pathways in each patient. In summary, inter-tissue SASP crosstalk converts the injured joint into a unified inflammatory network, providing multiple targets (cytokines, receptors, and immune modulators) for therapeutic intervention.

### Therapeutic strategies targeting senescent cells

Cell- and tissue-culture approaches provide controlled *in vitro* platforms to evaluate senolytic and senomorphic interventions. Precision therapies that eliminate senescent cells or suppress their deleterious secretions (“senotherapeutics”) are being explored as disease-modifying approaches to PTOA. Senescent-cell clearance can be achieved pharmacologically with senolytic drugs or *via* immune-mediated mechanisms, whereas SASP suppression can be achieved using senomorphic agents that dampen the secretory output of senescent cells. Recent pre-clinical studies have validated both strategies in musculoskeletal injury models.

Small-animal PTOA models further demonstrate the translational potential of these interventions *in vivo*. In a mouse model of PTOA, the senolytic combination of dasatinib and quercetin reduced SA-β-GALˆ+ senescent cells, improved cartilage integrity and attenuated synovitis. Likewise, intra-articular rapamycin acted as a senomorphic, lowering SASP factors and protecting cartilage after joint injury (Nogueira-Recalde et al., 2019).

Nanoparticle-based delivery systems offer enhanced precision and tissue penetration for delivering senotherapeutic agents. Specialized cationic lipid nanoparticles increase DNA uptake into chondrocytes while preventing nucleic-acid degradation, enabling delivery of senescence-modulating genes (Chaudhary et al., 2021). Design principles and carrier classes have continued to evolve to improve targeting efficiency and safety. Charge-reversal polymers enhance endosomal escape, and <100 nm polymer-stabilised particles distribute uniformly within cartilage under compression (Dey et al., 2021). Targeting strategies have increasingly focused on improving specificity while minimizing off-target exposure. Cartilage-homing peptides restrict off-target exposure. Metabolic cues also help folate-conjugated ZnO nanoparticles show ∼40% higher uptake in inflammatory senescent macrophages. Next-generation carriers have emerged as promising delivery platforms for senolytic agents. Innovations include ultrasmall (<10 nm) particles that directly ferry senolytic peptides (Panja & Jana, 2020) and cell-membrane-coated nanoparticles that “cloak” themselves with chondrocyte or macrophage membranes for immune evasion and joint homing (He et al., 2024). Therapeutic-window expansion has become an important goal for senotherapeutic nanocarriers. Such refinements widen the therapeutic window and cut systemic toxicity that limited earlier attempts. Nanodelivery also supports combination therapy: PEG-grafted lipid particles co-encapsulate CRISPR-Cas cargo and hydrophobic drugs (Ansari et al., 2023), sustaining release >48 h. Translational considerations—including dose and timing—are critical for balancing efficacy and safety. Heterogeneous resistance demands balanced efficacy and safety. Early-phase data elsewhere show incorrect dosing causes ∼30% of medication problems (Fernandes, Ferreira & Andrade, 2022), and excess dosing can flip compounds from protective to cytotoxic (Mishra et al., 2024). Biomarker-guided adaptive protocols and PK/PD modelling (Liang et al., 2024a) may individualise regimens, while dynamic interval dosing could delay resistance (Vakil & Trappe, 2021).

Monoclonal-antibody platforms provide highly specific and tunable effector functions for senescent-cell targeting. Monoclonal antibodies (mAbs) offer high specificity, long serum half-life and tunable effector functions (Di Micco, 2024). Senolytic mAbs trigger ADCC or CDC to ablate senescent cells. Antibody–drug conjugates (ADCs) couple cytotoxins to senescence-specific antibodies, achieving complete ablation of uPARˆ+ senescent fibroblasts *ex vivo* and now entering first-in-human dose-escalation (Ro et al., 2024). BiTEs redirect cytotoxic T cells to senescent targets with picomolar potency (Yang et al., 2023). Radio-immunotherapy using β-emitting anti-B2M antibodies reduced senescent-cell burden by 60% in aged mouse joints (Poblocka et al., 2021). Neutralising (senomorphic) antibodies against SASP drivers such as IL-1β or GDF-15 temper paracrine damage and are advancing clinically (Xiong et al., 2024).

Comparatively, senolytic mAbs provide definitive clearance but may provoke transient “senolytic crisis”, whereas senomorphic mAbs are safer but leave the senescent reservoir intact. In-silico optimisation of loading–maintenance regimens (Minichmayr et al., 2024) aim to balance efficacy and toxicity.

A summary of representative senotherapeutic strategies, their mechanisms, evidence, and developmental stages is presented in Table 2.

**Table 2:** Representative therapeutic strategies targeting senescent cells in PTOA and their development stage.

**Strategy category (matching main text sub-sections)**	**Representative agent(s)/platform**	**Anti-senescence mechanism**	**Key PTOA evidence**	**Development stage/main limitations**
Cell- and Tissue-Culture Approaches (Senolytics)	Dasatinib + Quercetin (D+Q)	BCR-ABL/Src inhibition + flavonol synergy; selectively induces apoptosis of senescent chondrocytes	Single intra-articular dose cleared p16ˆNK4aˆ+ cells and reduced cartilage loss in DMM mice (Jeon et al., 2017)	Pre-clinical proof-of-concept; off-target toxicity with systemic delivery
	Navitoclax (ABT-263)	BCL-2/BCL-xL inhibition; intrinsic apoptotic activation in SnCs	Weekly IA injections attenuated degeneration and improved gait in DMM rats (Li et al., 2018)	Pre-clinical; dose-limiting thrombocytopenia hampers systemic use
	Fisetin	Flavonol that disrupts pro-survival pathways in SnCs; antioxidant	Reduced SASP markers and pain in PTOA mice; Phase I knee-OA trial ongoing (NCT04770064)	Early clinical safety evaluation; efficacy yet to be proven
Cell- and Tissue-Culture Approaches (Senomorphics)	Tofacitinib (pan-JAK inhibitor)	Suppresses SASP via JAK/STAT blockade without killing senescent cells	*In vitro*: ↓ IL-6, MMP-13 in OA chondrocytes; *ex vivo* human cartilage	FDA-approved for RA; immunosuppression risk in chronic use
Small-Animal PTOA Models	Rapamycin	mTOR inhibition; SASP suppression and autophagy activation	Intra-articular rapamycin lowered SASP factors and preserved cartilage post-injury (Nogueira-Recalde et al., 2019)	Pre-clinical; systemic use limited by immunosuppression
Nanoparticle-Based Delivery Systems	Folate-ZnO NPs	Metabolic targeting of inflammatory senescent macrophages; enhanced uptake (∼40%)	Inflammatory senescent macrophages in PTOA models (Dey et al., 2021)	Pre-clinical; safety profile under evaluation
	PEG-grafted lipid NPs (CRISPR-Cas + hydrophobic drugs)	Co-delivery platform; sustained intra-articular release >48 h	PTOA-relevant nanodelivery proof-of-concept (Ansari et al., 2023)	Pre-clinical; manufacturing complexity
Monoclonal-Antibody Platforms	Anti-uPAR ADC	Antibody–drug conjugate	Complete clearance *ex vivo*; entering first-in-human dose-escalation (Ro et al., 2024)	Early clinical; potential “senolytic crisis”
	Anti-IL-1β mAb (Canakinumab)	Neutralises SASP driver cytokine IL-1β	ACLT rabbit knees: reduced MMP activity and cartilage erosion (Wang, Lankhorst & Bernards, 2022)	Phase III CV trials show safety; high cost; systemic dosing
Combined Regenerative + Anti-senescence	MSCs over-expressing α-Klotho	Paracrine anti-SASP signals; cartilage matrix repair	Rat PTOA model: restored cartilage thickness, ↓ p16ˆINK4aˆ staining (Zhang et al., 2025)	Pre-clinical; manufacturing & regulatory complexity

### Outstanding questions and future directions

Mechanistic gaps and model limitations: Although p16ˆINK4a-positive cell accumulation is well-documented in PTOA, the precise sequence linking mechanical overload to persistent senescence remains unclear.

**Figure 1:**
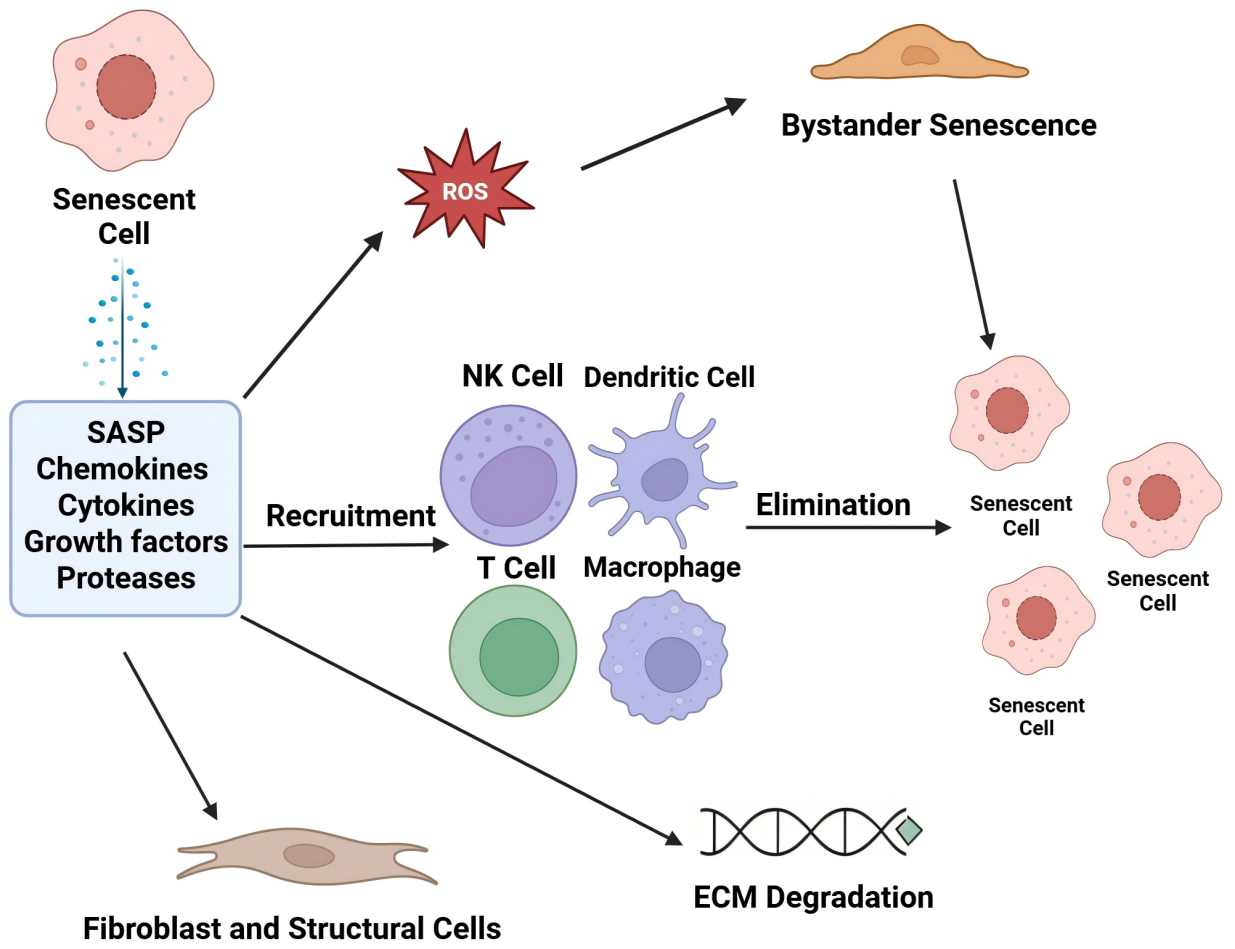
Cellular interplay following SASP activation. Created with BioRender.com.

Primary danger signals, such as DAMPs released from injured cartilage, initiate innate immune activation and drive inflammatory cascades in PTOA. Mechanical overload and micro-damage liberate damage-associated molecular patterns (DAMPs)—for example, aggrecan fragments, mitochondrial DNA, and extracellular ATP—from injured cartilage. These DAMPs promptly activate TLR2/4 on synovial dendritic cells and macrophages; robust TLR up-regulation has been documented in an iodoacetate-induced osteoarthritis model (Nie et al., 2019a; Nie et al., 2019b). The ensuing release of IL-1β, TNF-α, and reactive oxygen species amplifies NF-κB signalling in chondrocytes, driving p16/p21 induction and a full SASP response. The proposed cellular interactions following SASP activation are illustrated in Fig. 1.

The chronology of PTOA progression can be divided into acute, early, and intermediate phases. Acute phase (0–48 h). Cartilage overload ruptures chondrocyte membranes, sparks a burst of ROS, and induces DNA damage, activating p53-dependent p21 and transient NF-κB signalling (Dilley et al., 2023; Lieberthal, Sambamurthy & Scanzello, 2015). Early phase (1–4 weeks). Persistent oxidative stress plus DAMP-TLR engagement stabilises p16 expression in chondrocytes; the SASP peaks, with marked IL-1β, IL-6 and MMP-13 secretion (Jeon et al., 2017; Nie et al., 2019a; Nie et al., 2019b). Intermediate phase (1–3 months). VEGF-driven endothelial proliferation breaches the tidemark, while RunX2-positive osteoprogenitors accumulate in perivascular niches, coupling angiogenesis to subchondral bone sclerosis (Su et al., 2020; Hu et al., 2021). Chronic phase (>3 months). Ongoing NF-κB activation, osteophyte formation and cartilage–bone crosstalk consolidate structural degeneration; senescent-cell burden plateaus (Loeser, Collins & Diekman, 2016).

Most small-animal PTOA models rely on single-impact or surgical destabilization paradigms that do not capture repetitive sub-failure loading, multi-trauma, or patient-specific risk factors, such as obesity and diabetes (Dilley et al., 2023). Cartilage-only injury models overlook critical contributions from the subchondral bone, synovium, and sensory nerves, whereas large-animal studies remain scarce owing to cost and ethical constraints. Advanced *in-vitro* platforms are already beginning to bridge these gaps. Microphysiological ‘joint-on-chip’ systems are emerging; for example, a patient-specific microfluidic device that co-cultures cartilage and synovium replicates inflammatory crosstalk and allows personalised screening of orthobiologics (Petta et al., 2024). Similarly, the mechanically active uBeat^®^ MultiCompress OA-on-chip applies cyclic compression and shear to human cartilage microtissues, enabling evaluation of injectable therapeutics under native-like loading regimes (Palma et al., 2024). Complementary *ex-vivo* osteochondral platforms extend culture windows from days to weeks: long-term human tibial-plateau explants preserve cartilage and marrow viability for up to four weeks (Kleuskens et al., 2021), whereas a bovine osteochondral damage model that combines brief collagenase treatment with multiaxial loading recapitulates early inflammatory cascades relevant to overload-induced PTOA (Wen et al., 2025). Despite this progress, community-wide standards for benchmarking senescence read-outs and validating *in-vivo* correlates remain urgently needed.

Further research is needed to unravel how acute injury triggers long-lasting senescence signals and develop models that reflect the clinical complexity of PTOA. Integration of multi-omics and systems biology: Current PTOA datasets are dominated by transcriptomics; however, an integrative multi-omic approach (proteomic, metabolomic, and epigenomic) is needed to resolve cell type-specific senescence trajectories and identify combinatorial therapeutic targets. Single-cell multi-omics combined with spatial transcriptomics can map SASP heterogeneity across cartilage, synovium, and subchondral bone, whereas network-based modelling may reveal master regulators that coordinate inflammatory, metabolic, and mechanotransduction pathways (Wei et al., 2025; Segarra-Queralt, Piella & Noailly, 2023). Publicly accessible, longitudinal, and multi-omic repositories can facilitate meta-analyses and machine-learning approaches to predict individual PTOA risk and treatment responses. Embracing systems biology is crucial for understanding how various senescent drivers interact and for designing interventions that target multiple pathogenic nodes. Clinical trial design and personalized interventions: Early phase senolytic trials for musculoskeletal disorders must balance timing, intra-articular dosing, and off-target safety. Adaptive trial designs that stratify patients by “molecular age” (*e.g.*, synovial p16/p21 load or a panel of SASP proteins) could enrich likely responders and reduce sample sizes. Novel imaging endpoints that combine quantitative MRI with activatable senescence probes—such as NIR fluorophores that highlight senescent-cell niches—can reveal structural and cellular changes within months. β-Gal–activatable NIR probes like XZ1208 have already visualised senescent chondrocytes in DMM mouse knees (Hu et al., 2023). Similarly, a β-gal MRI contrast agent has recently detected senescent cells in porcine joints, thereby accelerating proof-of-concept studies (Nernekli et al., 2025). Precision rehabilitation protocols that titrate mechanical loading based on wearable sensor feedback should be accompanied by pharmacological interventions to avoid reinducing senescence in rejuvenated joints. Ultimately, personalized treatment algorithms guided by biomarkers and patient biomechanics are required to maximize efficacy and safety.

## Conclusions

Trauma-induced cellular senescence constitutes a unifying mechanism that links the mechanical, inflammatory, and metabolic sequelae of joint injury to the acceleration osteoarthritis development. Elucidating the spatiotemporal dynamics of senescent cell accrual and SASP propagation in PTOA has inspired the new generation of diagnostic probes and targeted therapeutics. However, successful translation requires refined preclinical models, multi-omic integration, and biomarker-guided clinical trials that align senolytic or senomorphic treatments with individual joint-loading profiles and systemic risk factors. By embedding these advances in an interdisciplinary framework spanning orthopaedics, geroscience, and bioengineering, the field has been poised to shift PTOA management from palliative symptom relief to mechanism-based disease modification. In the long term, targeting senescent cells could extend the functional lifespan of injured joints and improve the quality of life of millions of predominantly young and active patients.

## Additional Information and Declarations

### Data Availability

The following information was supplied regarding data availability: This is a literature review.
